# The genome sequence of field maple, *Acer campestre* L. (Sapindales: Sapindaceae)

**DOI:** 10.12688/wellcomeopenres.24795.2

**Published:** 2025-11-05

**Authors:** Maarten J. M. Christenhusz, Royal Botanic Gardens Kew Genome Acquisition Lab, Plant Genome Sizing collective, Darwin Tree of Life Barcoding collective, Wellcome Sanger Institute Tree of Life Management, Samples and Laboratory team, Wellcome Sanger Institute Scientific Operations: Sequencing Operations, Wellcome Sanger Institute Tree of Life Core Informatics team, Tree of Life Core Informatics collective, Darwin Tree of Life Consortium

**Affiliations:** 1 Royal Botanic Gardens Kew, Richmond, England, UK; 2 Curtin University, Perth, Western Australia, Australia

**Keywords:** Acer campestre, field maple, genome sequence, chromosomal, Sapindales

## Abstract

We present a genome assembly from an individual *Acer campestre* (the field maple; Tracheophyta; Magnoliopsida; Sapindales; Sapindaceae). The genome sequence is 565.1 megabases in span. Most of the assembly is scaffolded into 14 chromosomal pseudomolecules, in comprising 13 large chromosomes and one smaller B chromosome.. The mitochondrial and plastid genome assemblies have lengths of 769.91 kilobases and 156.29 kilobases in length, respectively. This assembly was generated as part of the Darwin Tree of Life project, which produces reference genomes for eukaryotic species found in Britain and Ireland.

## Species taxonomy

Eukaryota; Viridiplantae; Streptophyta; Streptophytina; Embryophyta; Tracheophyta; Euphyllophyta; Spermatophyta; Magnoliopsida; Mesangiospermae; eudicotyledons; Gunneridae; Pentapetalae; rosids; malvids; Sapindales; Sapindaceae; Hippocastanoideae; Acereae; *Acer*; *Acer campestre* L., 1753 (NCBI:txid66205).

## Background

The field maple, *Acer campestre* L., is a deciduous tree up to 25 m (but usually smaller) with corky bark and opposite, deeply lobed leaves. It flowers in spring with clusters of yellowish, sweet-scented flowers, followed after pollination by insects, by a pair of anemochorous winged seeds (samaras) in late summer. It is the only native maple in the UK and the only native member of the soapberry family, Sapindaceae. It is commonly found in hedgerows, in broadleaf forests or along edges of meadows across Europe, from southern Sweden to Spain and east to Poland, Iran and Uzbekistan. It is naturalised in Ireland, Portugal, western Russia, Kazakhstan, New Zealand and the USA ( POWO, 2024). In East Asia it is replaced by the closely related *Acer miyabei* Maxim., which occupies a similar ecological niche ( Grimm & Denk, 2014).

Field maple forms an intermediate stage in forest succession, germinating in the shade of other trees, while requiring more light when fruiting, but the relatively small and slow-growing trees are eventually outcompeted by larger tree species. It is usually found on neutral to alkaline soils, and is a common element of hedgerows and forest edges in much of Britain, although it never dominates the vegetation.

Because this species grow slowly and is generally small, it is not of major commercial interest. However, the white wood is strong and hard and can be used in carpentry, for wood turning and for wooden musical instruments ( Mills, 1996). The tree is also frequently used for planting in native hedgerow mixes and in rewilding and reforestation programmes.

The genetic structure across its distribution is the result of post-glacial recolonisation, but it also shows sharp latitudinal gradients, which are likely the result of considerable habitat fragmentation and limited seed dispersal ( Chybicki *et al.*, 2014; Wahlsteen *et al.*, 2023).

Flowers of field maple are bisexual, but individuals usually have complex temporal patterns of sex expression during the flowering season ( De Jong, 1976; Gleiser & Verdú, 2005). The species is therefore mostly allogamous, although individual trees are known to be capable of self-fertilisation ( Bendixen, 2001).

The high-quality genome of the field maple, *Acer campestre* joins the other whole genome sequences available for six other *Acer* species (e.g., Chen *et al.* 2023; Yu *et al*., 2021; Zhang *et al*. 2024) and will be invaluable for understanding more about the genetics underlying this native species, including establishing the genetic basis for the variation in floral sexual expression in this and related maples.

## Genome sequence report

**Figure 1.**
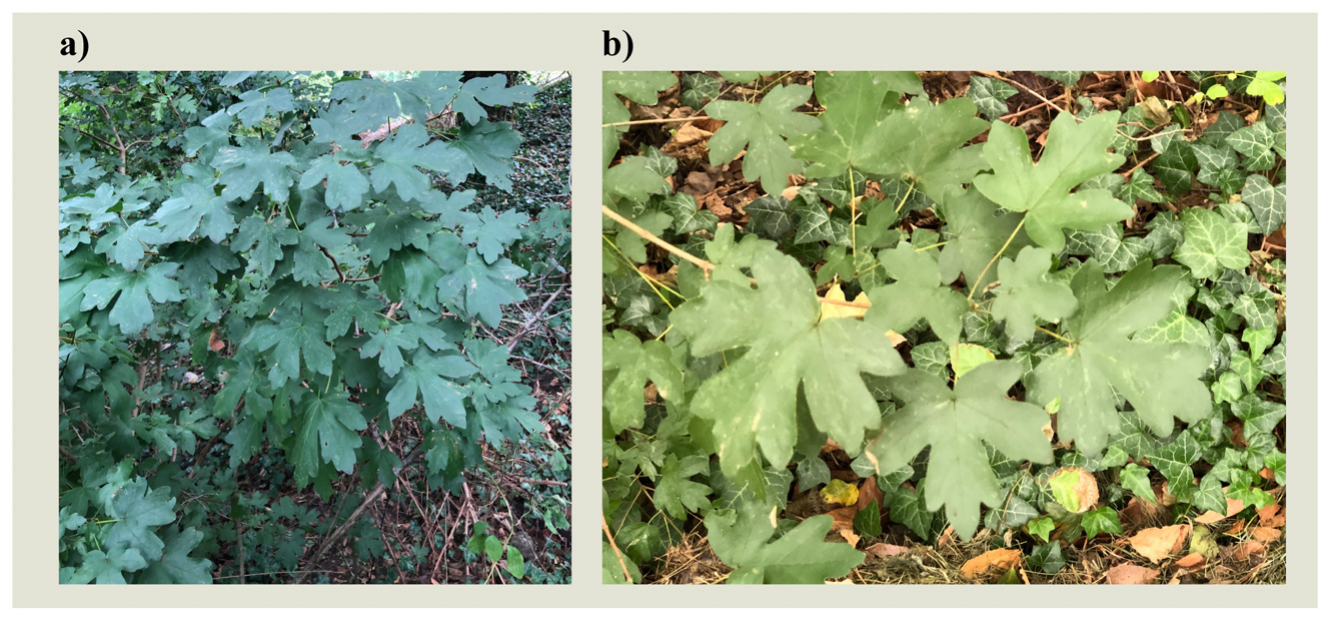
Photograph of the *Acer campestre* (drAceCamp1) specimen used for genome sequencing.

The genome was sequenced from a specimen of *Acer campestre* ( Figure 1) collected from Petersham Common, Richmond, Surrey, UK (51.45, –0.30). Using flow cytometry, the genome size (1C-value) was estimated to be 0.81 pg, equivalent to 790 Mb. A total of 41-fold coverage in Pacific Biosciences single-molecule HiFi long reads was generated. Primary assembly contigs were scaffolded with chromosome conformation Hi-C data. Manual corrections included 98 breaks, 84 joins, and removal of 127 haplotypic duplications, reducing the assembly length by 7.73% and the scaffold number by 31.11%, and increasing the scaffold N50 by 6.58%.

**Figure 2.**
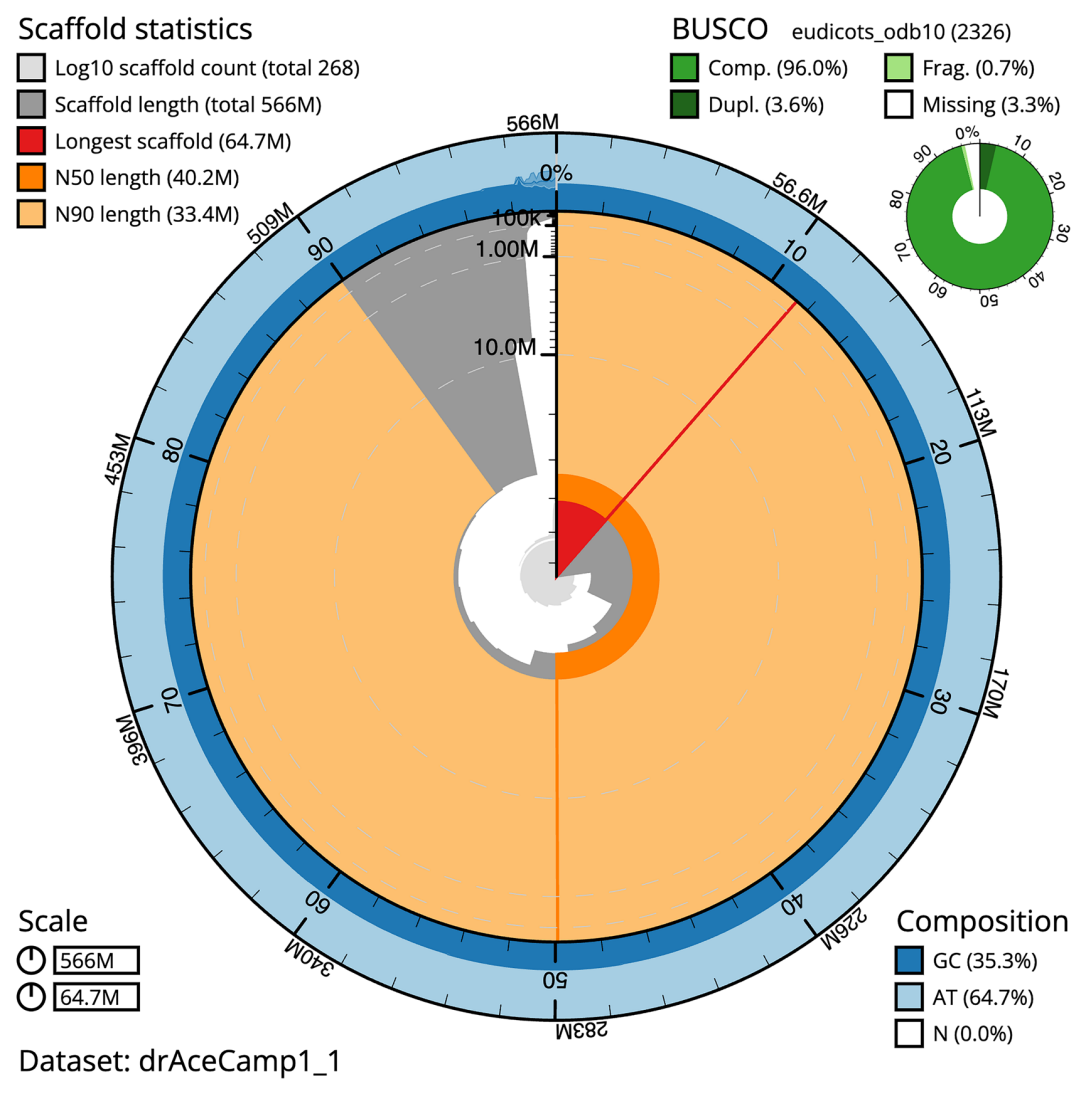
Genome assembly of *Acer campestre*, drAceCamp1.1: metrics. The BlobToolKit snail plot shows N50 metrics and BUSCO gene completeness. The main plot is divided into 1,000 bins around the circumference with each bin representing 0.1% of the 566,012,055 bp assembly. The distribution of scaffold lengths is shown in dark grey with the plot radius scaled to the longest scaffold present in the assembly (64,748,220 bp, shown in red). Orange and pale-orange arcs show the N50 and N90 scaffold lengths (40,156,598 and 33,405,154 bp), respectively. The pale grey spiral shows the cumulative scaffold count on a log scale with white scale lines showing successive orders of magnitude. The blue and pale-blue area around the outside of the plot shows the distribution of GC, AT and N percentages in the same bins as the inner plot. A summary of complete, fragmented, duplicated and missing BUSCO genes in the eudicots_odb10 set is shown in the top right. An interactive version of this figure is available at https://blobtoolkit.genomehubs.org/view/drAceCamp1_1/dataset/drAceCamp1_1/snail.

**Figure 3.**
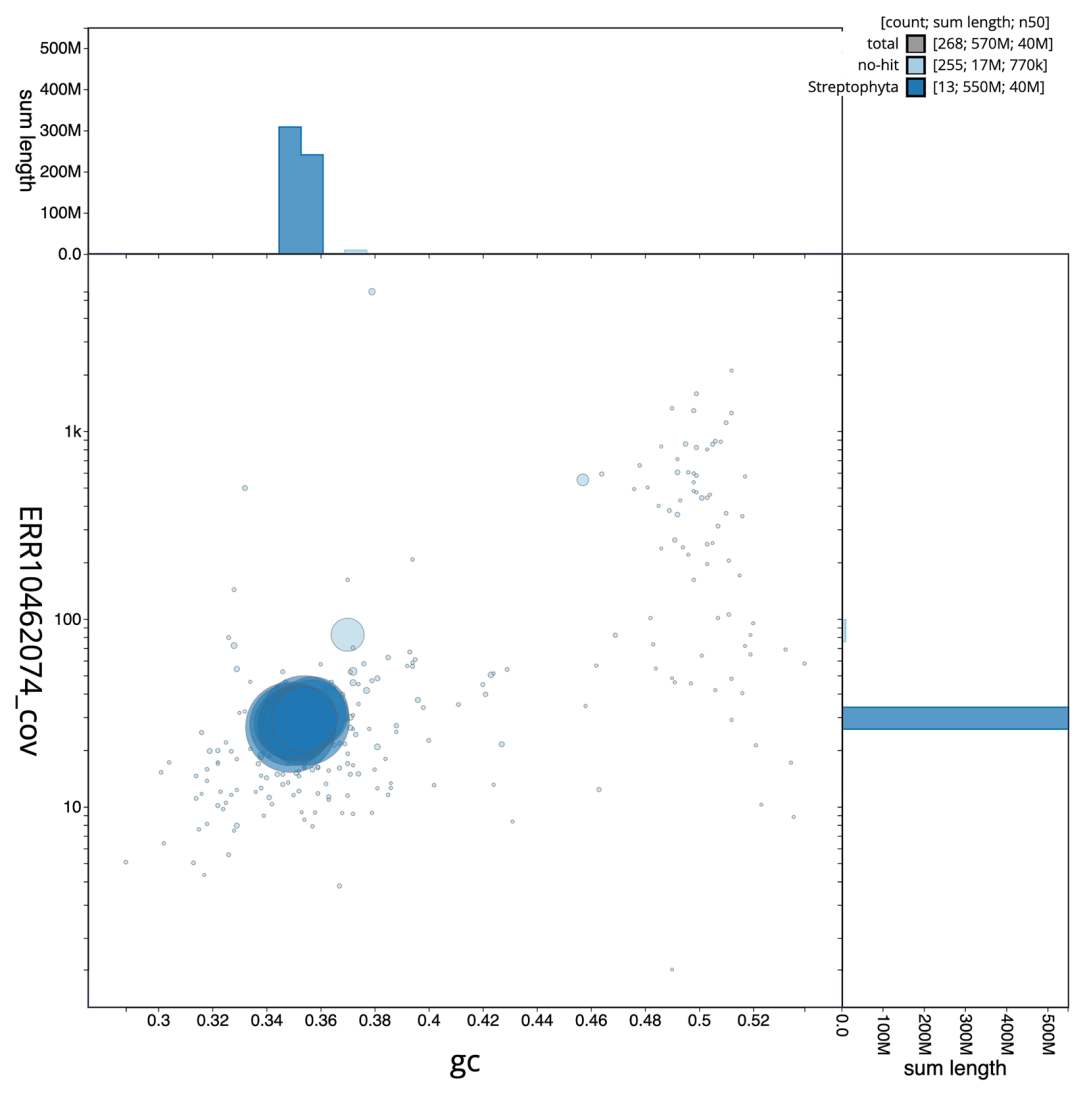
Genome assembly of *Acer campestre*, drAceCamp1.1: BlobToolKit GC-coverage plot. Scaffolds are coloured by phylum. Circles are sized in proportion to scaffold length. Histograms show the distribution of scaffold length sum along each axis. An interactive version of this figure is available at https://blobtoolkit.genomehubs.org/view/drAceCamp1_1/dataset/drAceCamp1_1/blob.

**Figure 4.**
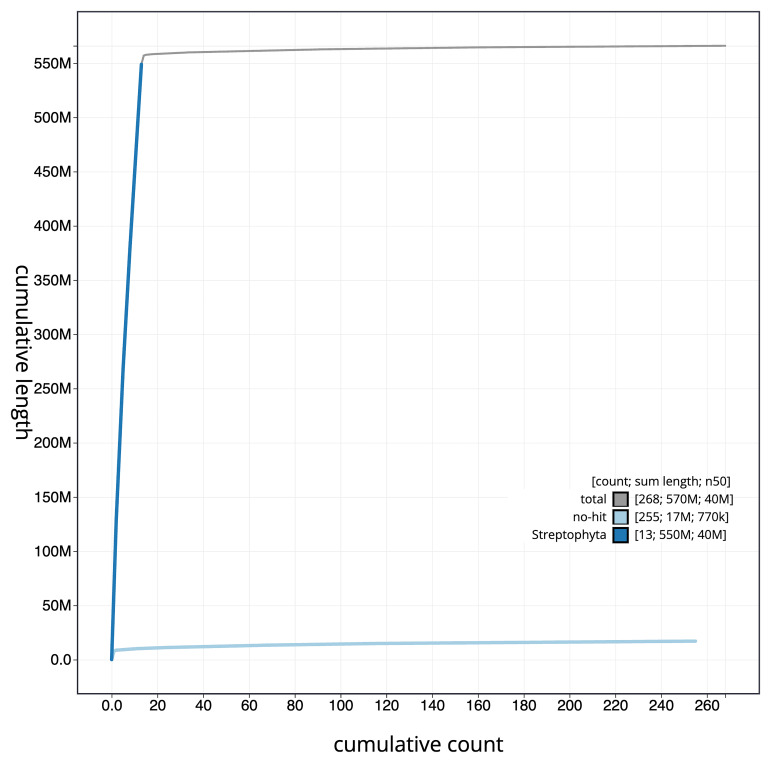
Genome assembly of *Acer campestre*, drAceCamp1.1: BlobToolKit cumulative sequence plot. The grey line shows cumulative length for all scaffolds. Coloured lines show cumulative lengths of scaffolds assigned to each phylum using the buscogenes taxrule. An interactive version of this figure is available at https://blobtoolkit.genomehubs.org/view/drAceCamp1_1/dataset/drAceCamp1_1/cumulative.

**Figure 5.**
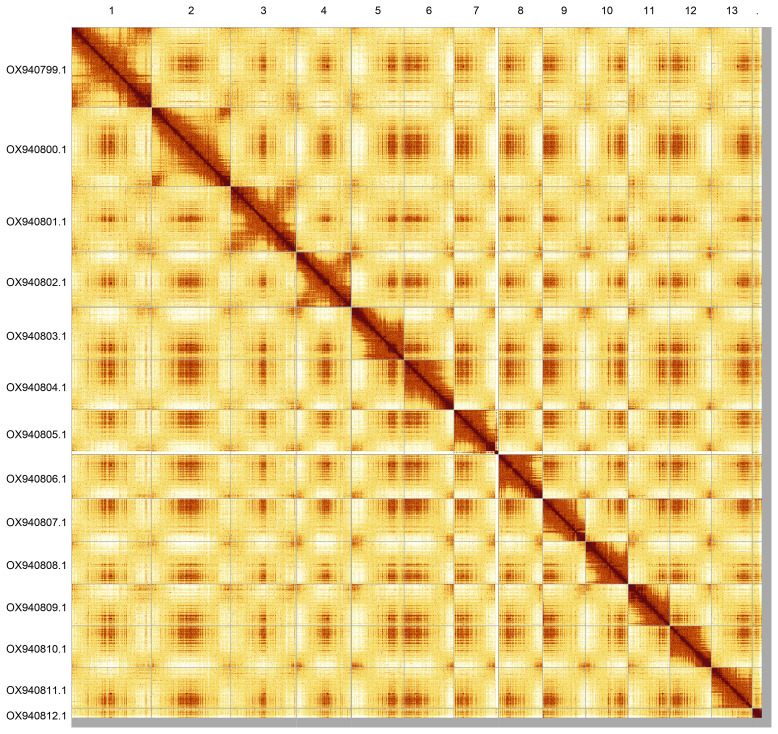
Hi-C contact map of the drAceCamp1.1 assembly. Assembled chromosomes are shown in order of size and labelled along the axes. The plot was generated using PretextSnapshot. An interactive version of this figure in HiGlass may be viewed at https://genome-note-higlass.tol.sanger.ac.uk/l/?d=PMA1zgWpQDSZISUbtMzcnQ.

The final assembly has a total length of 565.1 Mb in 266 sequence scaffolds with a scaffold N50 of 40.2 Mb ( Table 1). The snail plot in Figure 2 provides a summary of the assembly statistics, while the distribution of assembly scaffolds on GC proportion and coverage is shown in Figure 3. The cumulative assembly plot in Figure 4 shows curves for subsets of scaffolds assigned to different phyla. Most (98.54%) of the assembly sequence was assigned to 13 large chromosome-level scaffolds (ranging in size from 64.75–32.99 Mb) and 1 chromosome-level scaffold that was much smaller (7.93 Mb). Chromosome-scale scaffolds confirmed by the Hi-C data are named in order of size for chromosomes 1 to 13 ( Figure 5; Table 2). The smaller chromosome is a putative supernumerary B chromosome and is named B _1_ in Table 2. Alignments do not show homology of B _1_ with assembled chromosomes of *Acer yangbiense* (GCA_008009225.1) ( Yang *et al.*, 2019) and *Acer pseudosieboldianum* ( Li *et al.*, 2022), however. Chromosome 2 contains a heterozygous inversion at 18.7–21.2 Mb.

**Table 1.** Genome data for *Acer campestre*, drAceCamp1.1.

Project accession data		
Assembly identifier	drAceCamp1.1	
Species	*Acer campestre*	
Specimen	drAceCamp1	
NCBI taxonomy ID	66205	
BioProject	PRJEB57270	
BioSample ID	SAMEA7522523	
Isolate information	drAceCamp1: leaf tissue (DNA, Hi-C and RNA sequencing)	
Assembly metrics *		*Benchmark*
Consensus quality (QV)	Primary: 64.8; alternate: 64.9; combined: 64.9	*≥ 40*
*k*-mer completeness	Primary: 70.24%; alternate: 73.88%; combined: 98.28%	*≥ 95%*
BUSCO **	C:96.0%[S:92.3%,D:3.6%],F:0.7%,M:3.3%,n:2,326	*C ≥ 90%*
Percentage of assembly mapped to chromosomes	98.54%	*≥ 90%*
Organelles	Mitochondrial genome: 769.91 kb Plastid genome: 156.29 kb	*complete single* *alleles*
Raw data accessions		
PacificBiosciences SEQUEL II	ERR10462074, ERR10462075	
Hi-C Illumina	ERR10466809, ERR10466808	
PolyA RNA-Seq Illumina	ERR12035174	
Genome assembly		
Assembly accession	GCA_954870605.1	
*Accession of alternate haplotype*	GCA_954871055.1	
Span (Mb)	565.1	
Number of contigs	1366	
Contig N50 length (Mb)	0.8	
Number of scaffolds	266	
Scaffold N50 length (Mb)	40.2	
Longest scaffold (Mb)	64.75	

* Assembly metric benchmarks are adapted from column VGP-2020 of “Table 1: Proposed standards and metrics for defining genome assembly quality” from ( Rhie *et al.*, 2021). ** BUSCO scores based on the eudicots_odb10 BUSCO set using version 5.3.2. C = complete [S = single copy, D = duplicated], F = fragmented, M = missing, n = number of orthologues in comparison. A full set of BUSCO scores is available at https://blobtoolkit.genomehubs.org/view/drAceCamp1_1/dataset/drAceCamp1_1/busco.

**Table 2.** Chromosomal pseudomolecules in the genome assembly of *Acer campestre*, drAceCamp1.

INSDC accession	Name	Length (Mb)	GC%
OX940799.1	1	64.75	35.0
OX940800.1	2	63.81	35.5
OX940801.1	3	52.81	35.0
OX940802.1	4	44.4	35.0
OX940803.1	5	42.41	35.0
OX940804.1	6	40.16	35.5
OX940805.1	7	35.94	35.0
OX940806.1	8	35.52	35.5
OX940807.1	9	34.7	35.5
OX940808.1	10	34.37	35.0
OX940809.1	11	33.72	35.0
OX940810.1	12	33.41	35.5
OX940811.1	13	32.99	35.5
OX940813.1	MT	0.77	45.5
OX940814.1	Pltd	0.16	38.0
OX940812.1	B _1_	7.93	37.0

While not fully phased, the assembly deposited is of one haplotype. Contigs corresponding to the second haplotype have also been deposited. The mitochondrial and plastid genomes were also assembled and can be found as contigs within the multifasta file of the genome submission.

The estimated Quality Value (QV) of the final assembly is 64.8. The *k*-mer completeness is 70.24% for the primary assembly, 73.88% for the alternate haplotype, and 98.28% for the combined assemblies. The BUSCO v5.3.2 completeness of 96.0% (single = 92.3%, duplicated = 3.6%), using the eudicots_odb10 reference set ( *n* = 2,326).

## Methods

### Sample acquisition, genome size estimation and nucleic acid extraction

A specimen of *Acer campestre* (specimen ID KDTOL10103, ToLID drAceCamp1) was collected from Petersham Common, Richmond, Surrey, UK (latitude 51.45, longitude –0.30) on 2020-09-08. The specimen was collected and identified by Maarten J. M. Christenhusz (Royal Botanic Gardens Kew) and then frozen at –80 °C.

The genome size was estimated by flow cytometry using the fluorochrome propidium iodide and following the ‘one-step’ method as outlined in Pellicer *et al.* (2021). For this species, the General Purpose Buffer (GPB) supplemented with 3% PVP and 0.08% (v/v) beta-mercaptoethanol was used for isolation of nuclei ( Loureiro *et al.*, 2007), and the internal calibration standard was *Petroselinum crispum* ‘Champion Moss Curled’ with an assumed 1C-value of 2,200 Mb ( Obermayer *et al.*, 2002).

The workflow for high molecular weight (HMW) DNA extraction at the Wellcome Sanger Institute (WSI) includes a sequence of core procedures: sample preparation; sample homogenisation, DNA extraction, fragmentation, and clean-up. Protocols developed by the WSI Tree of Life core laboratory are publicly available on protocols.io ( Denton *et al.*, 2023).

In sample preparation, the drAceCamp1 sample was weighed and dissected on dry ice ( Jay *et al.*, 2023). For sample homogenisation, leaf tissue was cryogenically disrupted using the Covaris cryoPREP ^®^ Automated Dry Pulverizer ( Narváez-Gómez *et al.*, 2023). HMW DNA was extracted using the Automated Plant MagAttract v2 protocol ( Todorovic *et al.*, 2023a). HMW DNA was sheared into an average fragment size of 12–20 kb in a Megaruptor 3 system with speed setting 30 ( Todorovic *et al.*, 2023b). Sheared DNA was purified by solid-phase reversible immobilisation ( Strickland *et al.*, 2023), using AMPure PB beads to eliminate shorter fragments and concentrate the DNA. The concentration of the sheared and purified DNA was assessed using a Nanodrop spectrophotometer and Qubit Fluorometer and Qubit dsDNA High Sensitivity Assay kit. Fragment size distribution was evaluated by running the sample on the FemtoPulse system.

RNA was extracted from leaf tissue of drAceCamp1 in the Tree of Life Laboratory at the WSI using the RNA Extraction: Automated MagMax™ *mir*Vana protocol ( do Amaral *et al.*, 2023). The RNA concentration was assessed using a Nanodrop spectrophotometer and a Qubit Fluorometer using the Qubit RNA Broad-Range Assay kit. Analysis of the integrity of the RNA was done using the Agilent RNA 6000 Pico Kit and Eukaryotic Total RNA assay.

### Sequencing

Pacific Biosciences HiFi circular consensus DNA sequencing libraries were constructed according to the manufacturers’ instructions. Poly(A) RNA-Seq libraries were constructed using the NEB Ultra II RNA Library Prep kit. DNA and RNA sequencing was performed by the Scientific Operations core at the WSI on Pacific Biosciences Sequel II (HiFi) and Illumina NovaSeq 6000 (RNA-Seq) instruments. Hi-C data were also generated from leaf tissue of drAceCamp1 using the Arima2 kit and sequenced on the Illumina NovaSeq 6000, Illumina NovaSeq 6000 instrument.

### Genome assembly, curation and evaluation

Assembly was carried out with Hifiasm ( Cheng *et al.*, 2021) and haplotypic duplication was identified and removed with purge_dups ( Guan *et al.*, 2020). The assembly was then scaffolded with Hi-C data ( Rao *et al.*, 2014) using YaHS ( Zhou *et al.*, 2023). The assembly was checked for contamination and corrected using the gEVAL system ( Chow *et al.*, 2016) as described previously ( Howe *et al.*, 2021). Manual curation was performed using gEVAL, HiGlass ( Kerpedjiev *et al.*, 2018) and Pretext ( Harry, 2022). The mitochondrial and chloroplast genomes were assembled using MBG ( Rautiainen & Marschall, 2021) from PacBio HiFi reads mapping to related genomes. A representative circular sequence was selected for each from the graph based on read coverage.

To assess the assembly metrics, the *k*-mer completeness and QV consensus quality values were calculated in Merqury ( Rhie *et al.*, 2020). The genome was analysed within the BlobToolKit environment ( Challis *et al.*, 2020) and BUSCO scores ( Manni *et al.*, 2021; Simão *et al.*, 2015) were calculated. PretextSnapshot was used to generate a Hi-C contact map of the final assembly.

Table 3 contains a list of relevant software tool versions and sources.

**Table 3.** Software tools: versions and sources.

Software tool	Version	Source
BlobToolKit	4.1.7	https://github.com/blobtoolkit/blobtoolkit
BUSCO	5.3.2	https://gitlab.com/ezlab/busco
gEVAL	N/A	https://geval.org.uk/
HiCanu	2.2	https://github.com/marbl/canu
HiGlass	1.11.6	https://github.com/higlass/higlass
MBG	-	https://github.com/maickrau/MBG
Merqury	MerquryFK	https://github.com/thegenemyers/MERQURY.FK
MitoHiFi	2	https://github.com/marcelauliano/MitoHiFi
PretextView	0.2	https://github.com/wtsi-hpag/PretextView
purge_dups	1.2.3	https://github.com/dfguan/purge_dups
YaHS	2.1a-2	https://github.com/c-zhou/yahs

### Wellcome Sanger Institute – Legal and Governance

The materials that have contributed to this genome note have been supplied by a Darwin Tree of Life Partner. The submission of materials by a Darwin Tree of Life Partner is subject to the **‘Darwin Tree of Life Project Sampling Code of Practice’**, which can be found in full on the Darwin Tree of Life website here. By agreeing with and signing up to the Sampling Code of Practice, the Darwin Tree of Life Partner agrees they will meet the legal and ethical requirements and standards set out within this document in respect of all samples acquired for, and supplied to, the Darwin Tree of Life Project.

Further, the Wellcome Sanger Institute employs a process whereby due diligence is carried out proportionate to the nature of the materials themselves, and the circumstances under which they have been/are to be collected and provided for use. The purpose of this is to address and mitigate any potential legal and/or ethical implications of receipt and use of the materials as part of the research project, and to ensure that in doing so we align with best practice wherever possible. The overarching areas of consideration are:

•    Ethical review of provenance and sourcing of the material

•    Legality of collection, transfer and use (national and international)

Each transfer of samples is further undertaken according to a Research Collaboration Agreement or Material Transfer Agreement entered into by the Darwin Tree of Life Partner, Genome Research Limited (operating as the Wellcome Sanger Institute), and in some circumstances other Darwin Tree of Life collaborators.

## Data Availability

European Nucleotide Archive: *Acer campestre* (field maple). Accession number PRJEB57270; https://identifiers.org/ena.embl/PRJEB57270. The genome sequence is released openly for reuse. The *Acer campestre* genome sequencing initiative is part of the Darwin Tree of Life (DToL) project. All raw sequence data and the assembly have been deposited in INSDC databases. The genome will be annotated using available RNA-Seq data and presented through the Ensembl pipeline at the European Bioinformatics Institute. Raw data and assembly accession identifiers are reported in Table 1.

## Author information

Members of the Royal Botanic Gardens Kew Genome Acquisition Lab are listed here: https://doi.org/10.5281/zenodo.4786680.

Members of the Plant Genome Sizing collective are listed here: https://doi.org/10.5281/zenodo.7994306.

Members of the Darwin Tree of Life Barcoding collective are listed here: https://doi.org/10.5281/zenodo.4893703.

Members of the Wellcome Sanger Institute Tree of Life Management, Samples and Laboratory team are listed here: https://doi.org/10.5281/zenodo.16411243.

Members of Wellcome Sanger Institute Scientific Operations: Sequencing Operations are listed here: https://doi.org/10.5281/zenodo.14870789.

Members of the Wellcome Sanger Institute Tree of Life Core Informatics team are listed here: https://doi.org/10.5281/zenodo.10066637.

Members of the Tree of Life Core Informatics collective are listed here: https://doi.org/10.5281/zenodo.5013541.

Members of the Darwin Tree of Life Consortium are listed here: https://doi.org/10.5281/zenodo.4783558.
