# Supplementary material for: The genome sequence of field maple, Acer campestre L. (Sapindales: Sapindaceae)
Source: Wellcome Open Res. 2025 Nov 5;10:499. Originally published 2025 Sep 9. [Version 2] doi: 10.12688/wellcomeopenres.24795.2 (PMC12754365; doi:10.12688/wellcomeopenres.24795.2)
Supplement: Peer review history

# Reviewer report: Reviewer response for version 2

**DOI:** 10.21956/wellcomeopenres.27571.r141298

**Published:** 2025-12-31

**Authors:** Qionghou Li

**Affiliations:** 1 Nanjing Agricultural University, Nanjing, Jiangsu, China

This manuscript reports the sequencing, assembly, and quality assessment of the *field maple* genome, as well as the mitochondrial and chloroplast genomes. The authors used hifiasm to generate both primary and alternative assemblies and further anchored the contigs to chromosomes using Hi-C data, thereby achieving a chromosome-level assembly. The assembly quality is high, with good contiguity and completeness.

 The reported QV value above 60 is particularly impressive; however, it is quite common for QV estimates to be inflated when they are calculated using HiFi reads, as these are the same reads used for the assembly. I would prefer that Illumina reads be used to estimate the QV of the HiFi-based assembly to more reliably assess the base-level accuracy.

 Overall, the manuscript is well structured, clearly written, and the results are presented in an understandable way.

 I have only one minor concern: Hicanu appears in the “Software tools” section, but I did not find any corresponding step in the Methods where HiCanu was used. In addition, the version information for hifiasm is missing from the “Software tools” section.

Are sufficient details of methods and materials provided to allow replication by others?

Yes

Is the rationale for creating the dataset(s) clearly described?

Yes

Are the datasets clearly presented in a useable and accessible format?

Yes

Are the protocols appropriate and is the work technically sound?

Yes

Reviewer Expertise:

genome assembly, plant evolution

I confirm that I have read this submission and believe that I have an appropriate level of expertise to confirm that it is of an acceptable scientific standard.

# Reviewer report: Reviewer response for version 2

**DOI:** 10.21956/wellcomeopenres.27571.r141300

**Published:** 2025-12-30

**Authors:** Francesco Garassino

**Affiliations:** 1 University of Zurich, Zürich, Zurich, Switzerland

The report is clear and contains all essential information. I would've appreciated a little more information on the relevance of this genome in the *Acer* pool, but do not find it a limitation.

 The methodology is clear and well-documented. I'd suggest adding a direct link to the WSI DNA extraction.

 There are a number of small typos or grammar errors that I'd suggest fixing (e.g., "...  using the Arima2 kit and sequenced on the Illumina NovaSeq 6000, Illumina NovaSeq 6000 instrument." under "Sequencing", or "Because this species grow slowly and is generally small," under "Background"), so I'd suggest an additional full proofreading of the manuscript.

Are sufficient details of methods and materials provided to allow replication by others?

Yes

Is the rationale for creating the dataset(s) clearly described?

Yes

Are the datasets clearly presented in a useable and accessible format?

Yes

Are the protocols appropriate and is the work technically sound?

Yes

Reviewer Expertise:

Plant ecophyisiology, computational biology

I confirm that I have read this submission and believe that I have an appropriate level of expertise to confirm that it is of an acceptable scientific standard.

# Reviewer report: Reviewer response for version 2

**DOI:** 10.21956/wellcomeopenres.27571.r141302

**Published:** 2025-12-30

**Authors:** Sunil Kumar Sahu

**Affiliations:** 1 Key Laboratory of Genomics, Ministry of Agriculture, BGI Research, Shenzhen, China

This Data Note by Christenhusz et al. presents a high-quality, chromosome-level genome assembly for the field maple, *Acer campestre*. The authors have generated this genomic resource as part of the Darwin Tree of Life project. Using a combination of Pacific Biosciences HiFi long-read sequencing and Hi-C chromosome conformation capture, they have produced a 565.1 Mb assembly scaffolded into 14 pseudomolecules, corresponding to 13 autosomes and one putative B chromosome. The manuscript details the methodology for sample collection, sequencing, assembly, and curation, and presents key quality metrics (e.g., scaffold N50, BUSCO completeness, QV score) that demonstrate the assembly's high contiguity and accuracy. The authors rightly position this genome as a valuable resource for future studies on the genetics, evolution, and conservation of this ecologically important European tree species.

**Comments for the Authors**

 There are no major issues with this manuscript. The work is sound, and the conclusions regarding the quality of the dataset are well-supported by the evidence provided. 

1. In one sentence of abstract, "Most of the assembly is scaffolded into 14 chromosomal pseudomolecules, in comprising 13 large chromosomes and one smaller B chromosome," the word "in" is superfluous. It should read: "...pseudomolecules, comprising 13 large chromosomes...".
  My recommendation is to accept after **Minor Revision**.

Are sufficient details of methods and materials provided to allow replication by others?

Yes

Is the rationale for creating the dataset(s) clearly described?

Yes

Are the datasets clearly presented in a useable and accessible format?

Yes

Are the protocols appropriate and is the work technically sound?

Yes

Reviewer Expertise:

Plant genomics and evolution

I confirm that I have read this submission and believe that I have an appropriate level of expertise to confirm that it is of an acceptable scientific standard, however I have significant reservations, as outlined above.

# Reviewer report: Reviewer response for version 2

**DOI:** 10.21956/wellcomeopenres.27571.r141306

**Published:** 2025-12-30

**Authors:** Peng-Fe Ma

**Affiliations:** 1 Chinese Academy of Sciences, Kunming, China

The genome of the field maple ( *Acer campestre*) was sequenced and assembled in this study. In combination of HiFi long reads and Hi-C sequencing, a high quality  of chromosome-level assembly was obtained. Moreover, the assembly is relatively complete by evaluating the K-mer completeness and BUSCO. However, it is better to introduce some basic information on the karyotype of this species or the whole genus.

Are sufficient details of methods and materials provided to allow replication by others?

Yes

Is the rationale for creating the dataset(s) clearly described?

Yes

Are the datasets clearly presented in a useable and accessible format?

Yes

Are the protocols appropriate and is the work technically sound?

Yes

Reviewer Expertise:

Plant genomics and evolution

I confirm that I have read this submission and believe that I have an appropriate level of expertise to confirm that it is of an acceptable scientific standard.

# Reviewer report: Reviewer response for version 2

**DOI:** 10.21956/wellcomeopenres.27571.r141297

**Published:** 2025-12-30

**Authors:** Oliver Rupp

**Affiliations:** 1 Justus-Liebig-University, Giessengiessen, Germany

The authors present a genome report of *Acer campestre *(field maple).

 The genome sequencing was carried out using state-of-the-art methods, including PacBio HiFi and Hi-C sequencing. The primary assembly using hifiasm was cleaned and scaffolded using the Hi-C data and manually cleaned into chromosome level pseudomolecules. Data and assembly quality was shown with appropriate methods. Overall the methods are clearly presented and the quality of the data is shown.

 The pipeline and data are available at public databases and a gene annotation will be performed.

 Overall no major issues are to report.

Are sufficient details of methods and materials provided to allow replication by others?

Yes

Is the rationale for creating the dataset(s) clearly described?

Yes

Are the datasets clearly presented in a useable and accessible format?

Yes

Are the protocols appropriate and is the work technically sound?

Yes

Reviewer Expertise:

genome assembly and annotation

I confirm that I have read this submission and believe that I have an appropriate level of expertise to confirm that it is of an acceptable scientific standard.

# Reviewer report: Reviewer response for version 2

**DOI:** 10.21956/wellcomeopenres.27571.r140449

**Published:** 2025-12-11

**Authors:** Himanshu Saini

**Affiliations:** 1 Adama Science and Technology University, Adama, Ethiopia

The manuscript presents a clear and well-articulated rationale for generating the dataset(s), effectively demonstrating their relevance and value to the research community. The experimental and analytical protocols are appropriate, methodologically robust, and technically sound. Additionally, the authors provide sufficient detail regarding materials, procedures, and data processing steps to enable full reproducibility by other researchers. The datasets themselves are organized, clearly presented, and made available in an accessible and user-friendly format, ensuring they can be readily utilized for further analysis and future studies.

Are sufficient details of methods and materials provided to allow replication by others?

Yes

Is the rationale for creating the dataset(s) clearly described?

Yes

Are the datasets clearly presented in a useable and accessible format?

Yes

Are the protocols appropriate and is the work technically sound?

Yes

Reviewer Expertise:

Molecular Plant Biotechnology

I confirm that I have read this submission and believe that I have an appropriate level of expertise to confirm that it is of an acceptable scientific standard.
